# Signaling Modification by GPCR Heteromer and Its Implication on X-Linked Nephrogenic Diabetes Insipidus

**DOI:** 10.1371/journal.pone.0163086

**Published:** 2016-09-20

**Authors:** Hans K. H. Ng, Kaleeckal G. Harikumar, Laurence J. Miller, Billy K. C. Chow

**Affiliations:** 1 School of Biological Sciences, The University of Hong Kong, Hong Kong, China; 2 Department of Molecular Pharmacology and Experimental Therapeutics, Mayo Clinic, Scottsdale, Arizona, 85259, United States of America; Universita degli Studi di Bari Aldo Moro, ITALY

## Abstract

The involvement of secretin (SCT) and secretin receptor (SCTR) in regulating body water homeostasis is well established. Identified as one of the vasopressin (Vp)-independent mechanisms in fluid balance, SCT regulates aquaporin 2 (AQP2) in the kidney distal collecting duct cells through activating intracellular cAMP production. This ability to bypass Vp-mediated water reabsorption in kidney implicates SCT’s potential to treat nephrogenic diabetes insipidus (NDI). Research on NDI in the past has largely been focused on the searching for mutations in vasopressin receptor 2 (AVPR2), while the functional relationship between SCTR, AVPR2 and NDI remains unclear. Here, we demonstrate the interaction between SCTR and AVPR2 to modulate cellular signaling *in vitro*. Interestingly, we show in this report that upon heteromer formation with SCTR, R137H, a NDI-causing AVPR2 mutant that is defective in trafficking to cell surface, can functionally be rescued. Our data may provide an explanation for this clinically mild case of NDI, and insights into the pathological development of NDI in the future.

## Introduction

Water homeostasis is one of the most tightly regulated physiological events in the human body [1]. In addition to the well-recognized Vp axis, the existence of Vp-independent mechanisms in regulating water reabsorption is confirmed [2–12]. Among these, SCT was discovered to be a neurohypophysial factor secreted alongside Vp in the posterior pituitary to control fluid balance by stimulating Vp expression and release from the hypothalamic paraventricular nucleus [11]. SCT also stimulates water reabsorption in the kidney via activating the cAMP signaling pathway and subsequently AQP2 trafficking in the kidney distal collecting duct cells [12]. X-linked NDI is a form of NDI caused by AVPR2 gene mutation on the X chromosome, and the condition is characterized by very low urine osmolality plus marked increase in urine output [13]. Over 170 different mutations were discovered leading to various degree of impairment in kidney’s responsiveness to Vp stimulation [14]. There is no known cure for the disease; NDI patient management relies primarily on diuretics to reduce glomerular filtration rate, and supplemented by tightly controlled intake of sodium and water [13]. A handful of novel treatment strategies for NDI are currently under investigation. Notably, the vasopressin 1a receptor antagonist SR 49059 was reported as being effective [15, 16]. In the past, elucidation of the molecular mechanism of the disease focused heavily on AVPR2 [17, 18]. Most studies were based on clinical reports of AVPR2 mutations, followed by cloning and functional characterization of the mutants [19–21]. However, these studies were conducted in heterologous system expressing only the mutants [22–24]. There are recent evidences showing G protein-coupled receptors (GPCRs) function as monomer and oligomers, with oligomerization of GPCRs modulating a number of receptor physiologies from cellular signaling cascade to receptor trafficking [25, 26]. In light of SCT’s role in regulating body fluid, SCT was suggested as a potential treatment option [27]. As SCTR and AVPR2 are co-localized in the kidney distal tubules [10], in this report, we studied potential heteromer formation between SCTR with Vp receptors. We found that SCTR specifically hetero-oligomerizes with AVPR2, but not with AVPR1b. Both SCTR and AVPR2 primarily utilize the cAMP signaling pathway, but SCTR is also known to signaling through the calcium-IP3 pathway [28, 29]. We therefore investigated the functional consequences of receptor oligomer formation, and found that the interaction between SCTR and AVPR2 elicits differential receptor functions *in vitro*. Interestingly, we show here that upon heteromer formation with SCTR, R137H, a NDI-causing AVPR2 mutant that is defective in trafficking to cell surface, can functionally be rescued. Our data may provide an explanation for this clinically mild case of NDI, and insights into the pathological development of NDI in the future.

## Results

### *In vitro* BRET detection of mSCTR and mAVPR2 heteromer

**Fig 1:**
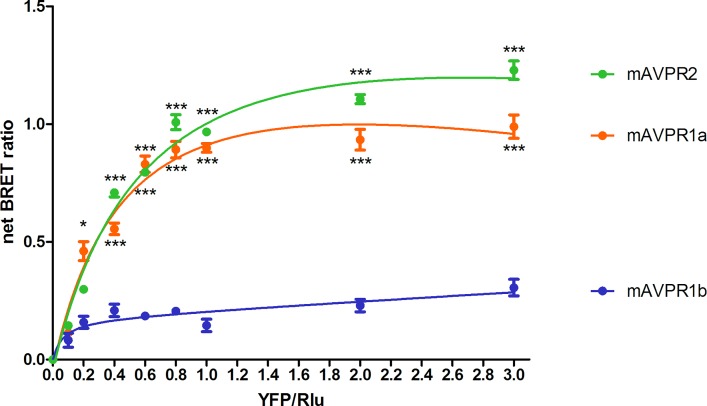
mSCTR specifically oligomerizes with mAVPR2, and mAVPR1a, but not mAVPR1b. Shown are the net BRET ratios for CHO-K1 cells expressing a combination of mSCTR-Rlu donor and mAVPR-YFP acceptor constructs. Saturable curves from BRET assays were obtained for mAVPR2 and mAVPR1a, but not for mAVPR1b. The data were mean±SEM from three to five independent experiments in triplicate. ***, P<0.001. **, P<0.01. *, P<0.05.

**Fig 2:**
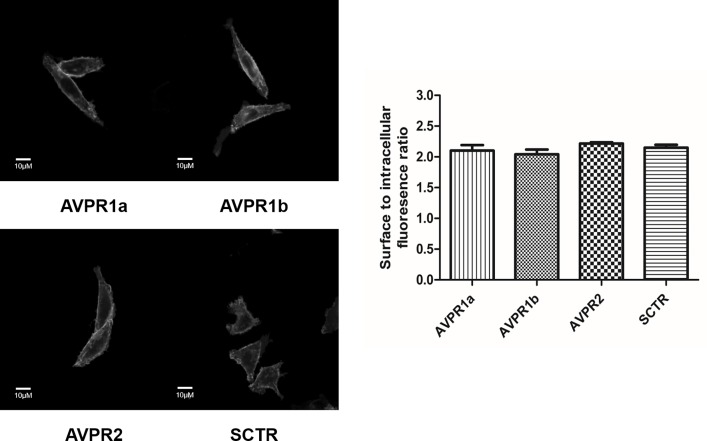
Surface expression of mAVPR1a, mAVPR1b, mAVPR2 and mSCTR are similar. Shown are representative images of CHOK1 cells expressing mAVPR1a/mAVPR1b/mAVPR2 or mSCTR constructs. Surface to intracellular fluorescence ratios were similar for these four types of cells. The data were mean±SEM from three independent experiments with 5–6 ROIs per sample. Scale bar, 10μM.

As previously documented, mSCTR co-expresses with mAVPR2 in the kidney tubular cells [10]. In this study, BRET saturation assay was used to confirm the specific heteromer formation of mSCTR with mAVPR2 and mAVPR1a, but not mAVPR1b, in transfected cells (Fig 1). This data provide evidence to show *in vitro* formation of specific GPCR heteromer formation between mSCTR/mAVPR2 and mSCTR/mAVPR1a. Confocal imaging and fluorescence intensity measurements were employed to ensure surface expression of the receptors were at comparable degree (Fig 2).

### mSCTR/mAVPR2 heteromer formation modifies receptor function

**Fig 3:**
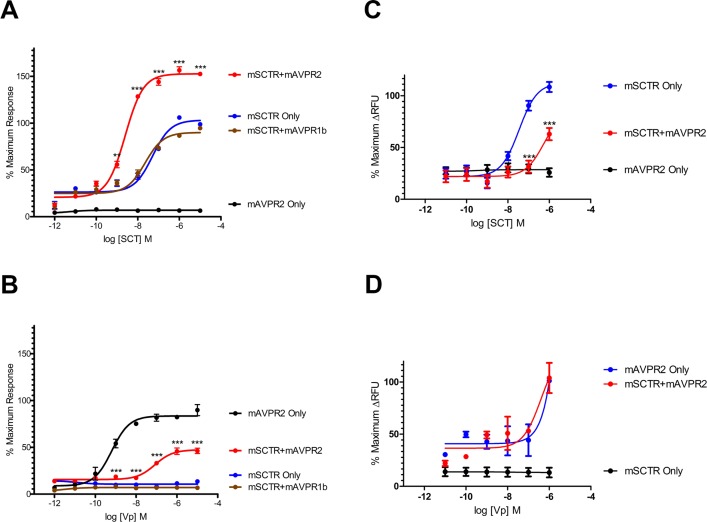
Signaling modification as a specific consequence of SCTR-AVPR2 oligomerization. Percentage changes in maximum cellular cAMP levels when cells were expressing a combination of mSCTR and/or mAVPR constructs, comparing to cAMP responses in cells bearing only the native receptor to the ligand. A) SCT stimulated a marked shift in E_max_ and potency in mSCTR in the presence of mAVPR2. No significant changes were observed when the non-interacting mAVPR1b replaced mAVPR2. B) Similarly, Vp stimulated a notable reduction in E_max_ and potency in mAVPR2 in the presence of mSCTR. The data were mean±SEM from three to five independent experiments in triplicate. ***, P<0.001. **, P<0.01. *, P<0.05. 10pM to 1μM SCT (panel C) or Vp (panel D) were treated to cells with mSCTR and/or mAVPR2. Calcium response curves are presented as percentages of maximal changes in RFU of cells expressing mSCTR or mAVPR2 only and stimulated with 1μM peptide. Except for cells with mSCTR only, a marked increase in cellular calcium response can only be seen at 1μM peptide concentration. Data were obtained from three individual experiments with 5–6 ROIs per dose.

**Fig 4:**
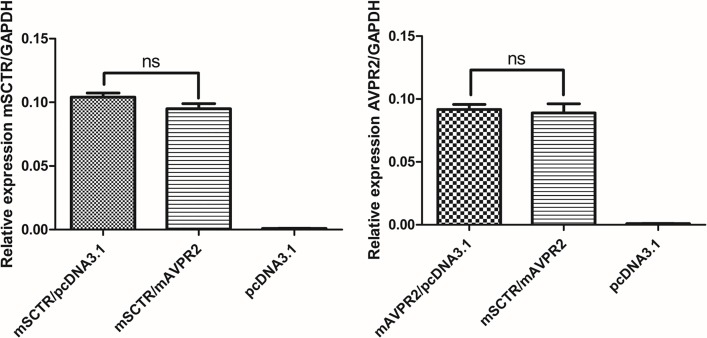
Comparable transcript levels of mSCTR and mAVPR2 in CHOK1 cells transiently transfected with combinations of mSCTR and mAVPR2 by quantitative real-time PCR. The total receptor gene transcript levels were compared to the internal house-keeping control GAPDH by the 2 ΔΔ ct method. There is no significant difference among the groups. mSCTR/pcDNA3.1, 1ug mSCTR plasmid with 1ug pcDNA 3.1 empty vector; mSCTR/mAVPR2, 1ug receptor plasmid each; pcDNA3.1, 2ug pcDNA3.1; mAVPR2/pcDNA3.1, 1ug mAVPR2 plasmid with 1ug pcDNA 3.1. Data are presented as means ± SEM from three independent experiments in duplicate. ns, not significant.

When co-expressing mSCTR and mAVPR2 *in vitro*, upon stimulation of a graded concentration of SCT (1 pM to 10 μM), SCT-induced cAMP production was potentiated (Fig 3A; E_max_ from 98.75 ±0.72 to 152.50±0.29% and EC_50_ from 51.44±9.92 to 2.49±0.25 nM), while no significant changes were found in control cells co-expressing mSCTR and mAVPR1b. In contrast, for Vp-induced cAMP (Fig 3B), co-expression of mSCTR reduced E_max_ from 89.75±5.92 to 46.23±2.88% and EC_50_ shifted from 0.64±0.15 to 84.20±21.80 nM. For intracellular calcium response, cells transfected with mSCTR showed a typical sigmoidal response curve (E_max_ = 108.46±4.84%, EC_50_ = 34.95±10.93 nM) to SCT. However, in the presence of mAVPR2, the calcium signals were mostly abolished (Fig 3C). In mAVPR2-transfected cells (Fig 3D), a response was observed only at 1 μM Vp, while the presence of mSCTR had no significant effect on maximal response nor potency. Quantitative RT-PCR data also suggest a comparable amount of receptor transcripts when cells were transfected with one or both receptors (Fig 4). This data support that signaling modification observed is due to heteromer formation and not an unbalance amount of receptors on the cell surface.

### Functional rescue of mAVPR2-R137H by co-expression with mSCTR

**Fig 5:**
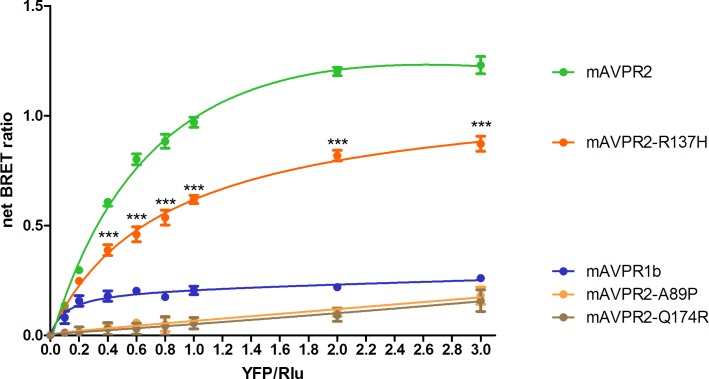
Oligo-complex formation rescues the constitutively endocytosed mAVPR2 mutant R137H. The constitutively β-arrestin bound R137H mutant of mAVPR2 is largely endocytosed. With co-expression of mSCTR, however, an increase in net BRET signal suggests rescue of the R137H mutant to the cell surface by heterocomplex formation. The mutations of A89P or Q174R, which lead to improper folded, non-surface reaching receptors, cannot be rescued by the formation of heteromer. Significance level was calculated against the mAVPR1b control. The data were mean±SEM from three to five independent experiments in triplicate. ***, P<0.001.

**Fig 6:**
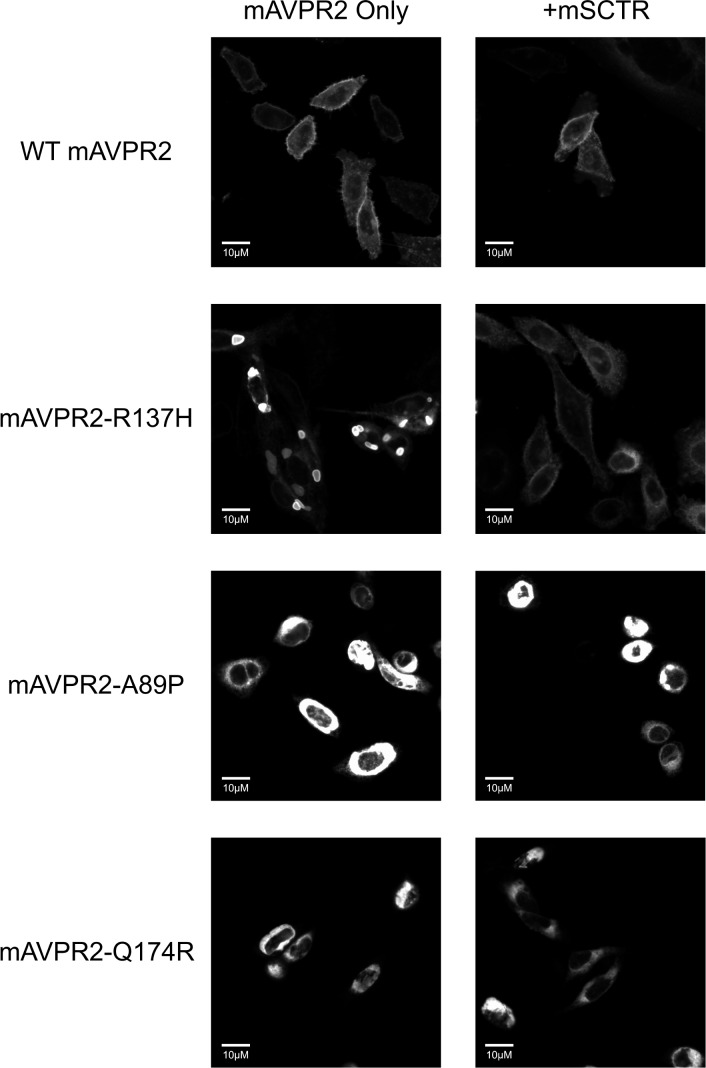
Rescue of the constitutively endocytosed R137H mAVPR2 mutant upon co-expression of mSCTR. Representative confocal images indicating the cellular location of mAVPR2-YFP receptors. The WT mAVPR2 is evenly distributed in the cell surface regardless of mSCTR co-expression. The R137H mutant is predominantly located in the intracellular endocytotic vesicles. Vesicular retention is not observed when mSCTR is co-transfected. The A89P and Q174R mutants cannot be rescued by mSCTR co-expression and remain intracellular. Scale bar, 10μM.

**Fig 7:**
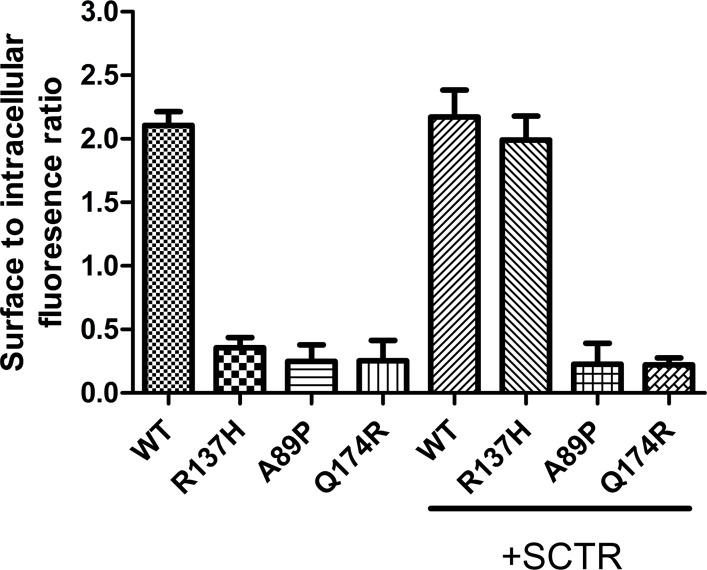
Surface to intracellular fluorescence ratio for cell expressing WT/mutant AVPR2 with or without SCTR co-expression. The presence of SCTR increased the amount of fluorescence on the cell surface in cell expressing R137H AVPR2 tagged with YFP, indicating a rescue of the mutant receptor. The data were mean±SEM from three independent experiments from 5–6 ROIs per sample.

**Fig 8:**
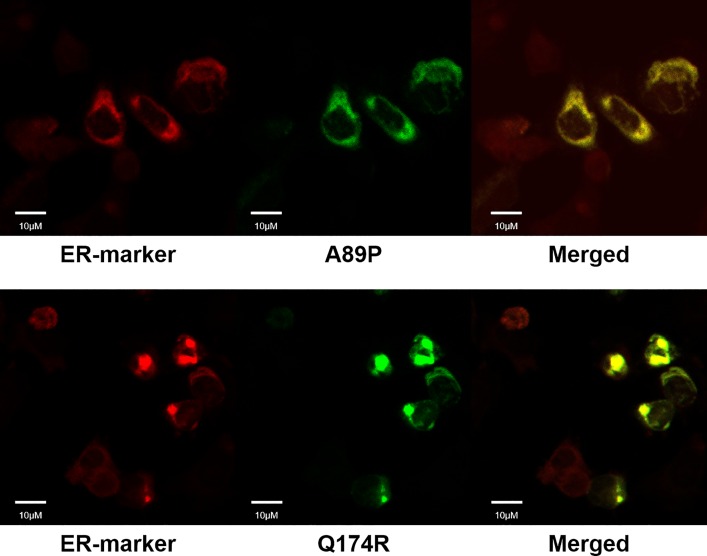
Localization of A89P/Q174R mutant within the ER. Using the ER specific marker Anti-calreticulin antibody conjugated with Alexa Fluor® 488, the A89P and Q174R AVPR2 mutants were determined to be resided within the ER. Scale bar, 10μM.

**Fig 9:**
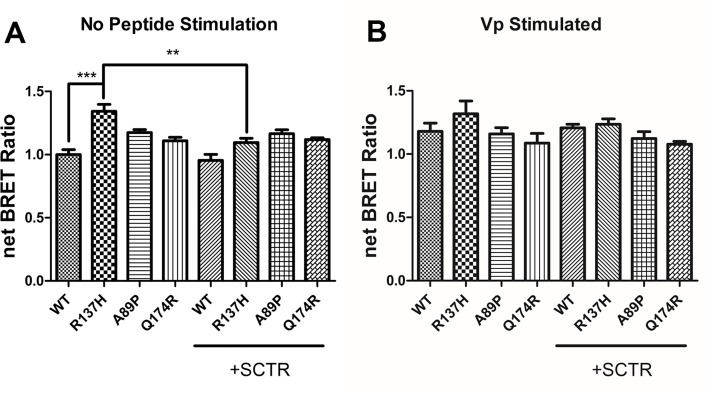
Rescue of R137H mutant by SCTR as reflected by reduced affinity to β-arrestin. The affinity between WT or mutant AVPR2 with β-arrestin were determined by BRET, using AVRP2 tagged with Rlu and β-arrestin tagged with YFP. A) In the native state, the R137H mutant shows significantly higher affinity to β-arrestin than the WT AVPR2. While the two other mutants, A89P and Q174R, showed slightly higher BRET than the WT receptor, but the increase was not significant. Upon co-expression of SCTR, β-arrestin affinity of R137H was significantly reduced compared to the scenario when no SCTR was present. B) BRET was measured at 10 min after addition of 1μM Vp to stimulate receptor internalization. WT AVPR2 showed increase affinity to β-arrestin, but not the R137H mutant without SCTR co-expression. With SCTR, R137H demonstrated increased β-arrestin binding, suggesting functional rescue of the receptor. Data are presented as means±SEM from three independent experiments in duplicate. ***, P<0.001, **, P<0.01.

**Fig 10:**
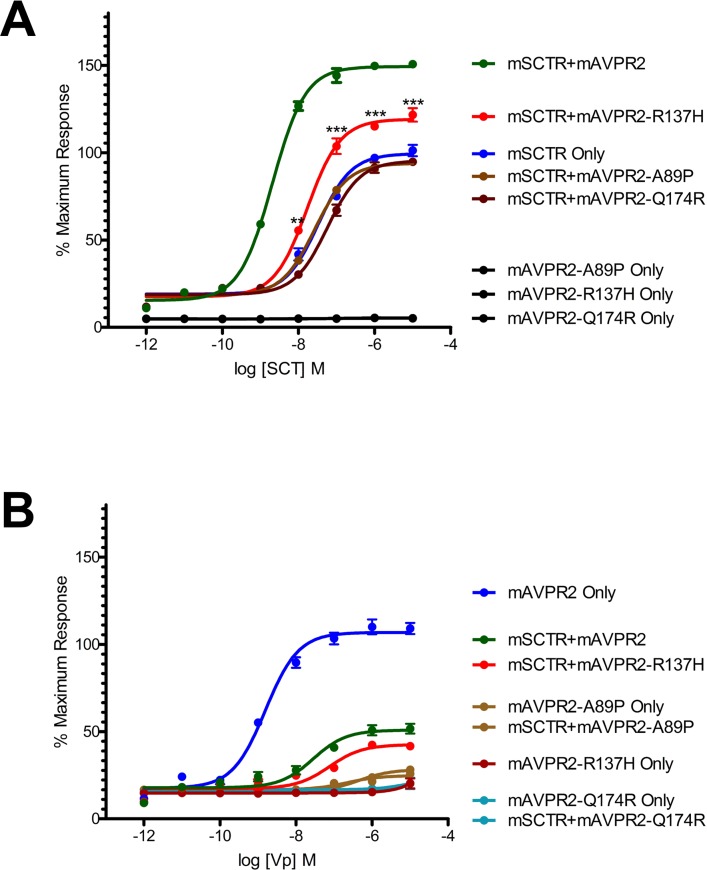
mSCTR-rescued mAVPR2-R137H functions similarly to WT receptor. In both SCT (panel A) and Vp (panel B) events, mSCTR was able to rescue the functioning of the mutated mAVPR2-R137H. Aligned with WT AVPR2, rescued AVPR2 mutant could potentiate SCT-induced cAMP. Note that although oligomer formation restores Vp’s ability in stimulating the R137H receptor, the effect of oligomer formation blunting cAMP response elicited by Vp was still in place. The data were mean±SEM from three to five independent experiments in triplicate. ***, P<0.001. **, P<0.01.

The R137H mutant of AVPR2 is irresponsive to Vp as it constitutively binds to β-arrestin [15, 16, 21, 23, 24, 30–32], and as a result, this mutant resides in endocytotic vesicles [33]. Although this mutant is non-functional, previous reports have associated it only with a mild form of NDI [16, 21, 32, 33]. In consideration of the physical association of mSCTR and mAVPR2 *in vitro*, the effects of co-expressing mSCTR with mAVPR2 mutants were studied. In addition to R137H, two other mutants, A89P and Q174R, that form misfolded proteins incapable of reaching the cell surface, were used as negative controls [34]. By BRET, mSCTR forms heteromer with R137H, but not with A89P nor Q174R (Fig 5). In addition, confocal microscopy was employed to visualize surface expression of receptors (Fig 6), with surface to intracellular fluorescence signals calculated (Fig 7). Cells were transfected with wild type (WT) or mutated form of mAVPR2-YFP, in the presence or absence of mSCTR. For WT, fluorescent signals were observed evenly throughout the cell surface independent to mSCTR expression. R137H mutant was found predominately in endocytotic vesicles characteristic to its constitutive binding to β-arrestin. However, upon co-expression with mSCTR, R137H was able to present to the cell surface. On the other hand, A89P and Q174R mutants were trapped in the cytoplasm with or without mSCTR. As an additional control to confirm the localization of A89P and Q174R mutants within the ER, immunofluorescence was performed using antibody targeting the ER specific marker calreticulin, conjugated with Alexa Fluor® 488 (Fig 8). In order to assay for the affinity between WT/mutant receptors with β-arrestin, BRET studies were performed using Rlu- tagged AVPR2s and YFP tagged β-arrestin. In native state, the R137H mutant showed a significantly higher affinity to β-arrestin, comparing to the WT AVPR2. However, such affinity can be significantly reduced when SCTR is coexpressed. The other forms of mutants could not be rescued by SCTR co-expression (Fig 9A). As a positive control, 1 μM Vp was treated to the cells and BRET measured 10 minutes after peptide stimulation (Fig 9B). As part of the internalization process, β-arrestin binding to the receptors resulted in an increase in BRET signal for the WT AVPR2, but not in the other mutants that were not functional. Nonetheless, with SCTR co-expression, the R137H mutant showed increased β-arrestin compared to the no peptide situation. This suggest also the rescue of the receptor to the cell surface and hence the correct functioning of the receptor. Combing the BRET data and the confocal images showing the surface expression of receptor, it suggests that R137H, in the presence of SCTR, can be presented to the cell surface. On top of rescuing R137H to the cell surface, such surface expression brought about the partial functioning of the receptor in response to both SCT and Vp stimulation. The signaling modifications in WT AVPR2 was found also in the SCTR-rescued R137H mutant (Fig 10).

## Discussion

The signaling modification observed in the present study illustrates the importance of GPCR heteromer in affecting cellular physiology. More notably, our data exemplifies a second instance of cross-class GPCR heteromer formation in addition to the previously elucidated SCTR-AT1aR heteromer [35]. Since both AT1aR and AVPR2 pose key roles in regulation of body water, heteromer formation with these receptors implicates SCTR as an important partner in fluid balance.

Functional rescue studies of the R137H mutant of AVPR2 were exclusively based on pharmacological chaperone [15, 16], and *in vitro* studies of the mutant were performed in the absence of SCTR [16, 21, 32, 33]. NDI is a rare disease but can be caused by over 170 of mutations in AVPR2 [14], clinical information is scarce, especially those specifying R137H mutation. In a previous report, the R137H patient showed distinctive readings for urine volume and urine osmolality compared with W164S and 185_193del patients [15]. Baseline urine volume and osmolality for the R137H patient were 125ml/30min and 200mOsm/kg respectively, while the two other patients were at 500ml/30min, 70mOsm/kg and 350ml/30min, 70mOsm/kg, respectively. This discrepancy in clinical parameters was noticed also by the authors, but no explanation was given. In another report involving two 8-year-old boys [31], urine output and osmolality for delG102 were 10–12 L/day and 100 mOsm/kg, and for R137H were >7L/day and 122mOsm/kg. It is difficult to compare various reports regarding the R137H mutant [15, 31, 33], due to age differences in patients and that not all relevant clinical figures (weight, plasma osmolality, plasma glucose content) were given. However, with the textbook definition of complete NDI having an urine osmolality of <100mOsm/kg [13], together with the general observation of elevated urine production in NDI caused by other mutations [31, 33, 34, 36, 37]. The R137H mutation paradoxically causes a less severe case of NDI, and our data here provide plausible explanations to this illusive situation. Concurrent with previous reports suggesting SCT and SCTR playing an important role in the water balance axis, our findings consolidate this idea. In the past, polypeptide fragment of AVPR2 and AVPR1a antagonist SR 49059 were reported as effective agents in rescuing AVPR2 mutants [15, 16, 38, 39], the current study suggests SCTR chaperone or SCT analogues as novel treatment alternatives for some forms of NDI.

## Methods

### Receptor constructs

The donor protein in bioluminescence resonance energy transfer (BRET) assay, mouse SCTR tagged at the carboxyl-terminus with *Renilla* luciferase construct (mSCTR-Rlu) was the generous gift of Prof. L.J. Miller from previous study [35]. Mouse AVPR 1a, 1b and 2, as well as β-arrestin (mAVPR1a, mAVPR1b, and mAVPR2; β-arrestin) were tagged at carboxyl end with yellow fluorescent protein (YFP) by cloning the respective cDNA into the vector pEYFP-N1 (Promega, Fitchburg, WI) as acceptor proteins in BRET (mAVPR1a/AVPR1b/AVPR2-YFP). Untagged mSCTR, mAVPR1a, mAVPR1b, and mAVPR2 were cloned into the vector pcDNA3.1(+). mAVPR2 mAVPR2 mutants were generated by site-directed mutagenesis using the QuikChange II Site-Directed Mutagenesis Kit (Agilent Technologies, Santa Clara, CA). The primers were designed using the online QuikChange Primer Design program (Agilent Technologies). The Rlu-tagged AVPR2 mutants were obtained by cloning the receptor into the Rlu-plasmid.

### Cell culture and transfection

CHO–K1 cells were purchased from ATCC (Manassas, VA). Cells were propagated in Minimum Essential Medium (MEM), pH 7.3 (Gibco, CA) supplemented with 10% (v/v) fetal bovine serum (FBS) (Gibco) in presence of 1% (v/v) penicillin G (100 U/ml) and streptomycin (100 μg/ml) (Invitrogen, CA), cultured at 37°C with 5% CO_2_ (Linde, HK). For conducting BRET and cAMP experiments, cells were plated on 6-well tissue culture plates (Nunc) at a density of 3×10^5^ cells per well 24 hrs before transient transfection. For calcium assays and confocal imaging, cells were seeded on 35mm glass bottom dishes coated with poly-d-lysine (MatTek Corporation, Ashland, MA) at a density of 1×10^5^ cell per well 24 hrs before transfection. Transfection of cells was done using the Viafect^TM^ reagent (Promega) according to manufacturer’s recommendation.

### Quantitative Real Time PCR

Quantitative real time PCR experiments were performed using Taqman reagents according to manufacturer’s protocol (Invitrogen). Gene transcript levels were compared to the internal house-keeping control GAPDH by the 2 ΔΔ ct method. The probes were as follows, SCTR: Mm1290794_m1; AVPR2: Mm00517071_m1.

### BRET assays

For saturation BRET assays, 1μg of the donor mSCTR-Rlu construct was transfected with graded amount (0.0–3.0μg) of acceptor constructs. An appropriate amount of pcDNA3.1 empty vector was added to maintain the total amount of DNA transfected be 4μg in all assays. BRET assays were performed 48 hrs after transfection. Cells were lifted using the non-enzymatic cell dissociation reagent Versene (Invitrogen) and washed in Hanks’ Buffered Saline Solution (HBSS, Invitrogen). After counting using an automated cell counter (LUNA; Logos Biosystems, Inc., S. Korea), 100,000 cells were added to each well of a black 96-well test plate (SPL life sciences, S. Korea). *Renilla* luciferase substrate Coelenterazine-h (Promega) was added to each well to a final concentration of 5μM. Bioluminescence emission was immediately measured at 440–500 nm (luciferin) and 510–590 nm (YFP) using a VICTOR X4 Multilabel Plate Reader (PerkinElmer, Inc., Waltham, MA). BRET ratios were calculated as long (510–590) / short (440–500) emission signals. Net BRET ratio was the BRET ratio of experimental group minus the BRET ratio of the negative control which expressed donor molecule only.

### cAMP assays

Cells were transfected with a combination of non-tagged receptors, at 1μg each. cAMP assay was performed 48 hrs after transfection using the LANCE cAMP kit (PerkinElmer) according to manufacturer’s protocol. Dose-dependent cAMP responses were assayed by treatment of mouse SCT (GenScript) or mouse Vp (Phoenix Pharmaceuticals, Inc., Burlingame, CA) at concentrations from 1 pM to 10 μM for 30 mins. Basal cellular cAMP was measured without peptide treatment. The Time-Resolved Fluorescence signal was detected in Victor X4 (PerkinElmer).

### Calcium assays

Transfected cells having 1μg each of non-tagged receptors were rinsed twice in solution α (HBSS with 2.5mM probenecid, 250mM NaOH, adjusted to pH 7.4 by HCl) 24 hrs after transfection. Loading of cells with cell permeant Fluo-4, AM (Invitrogen) was done at 1 μM Fluo-4, AM diluted in solution α containing 0.003% Pluronic® F-127 (Invitrogen) for 30 mins at room temperature. The cells were rinsed twice in solution α and allowed to incubate at room temperature for 30 mins before fluorescence signal was monitored. Fluorescence signal was measured on an LSM 710 NLO Confocal Laser Scanning Microscope (Carl Zeiss Microscopy GmbH, Jena, Germany) using a Plan-Neofluar 20x/0.50 Ph2 objective. The machine was set to excite the samples through an argon laser (LASOS Lasertechnik GmbH, Jena, Germany) at 488 nm and record 493–622 nm emission in a time series manner. The pinhole was set at 44μm and pixel dwell at 1.58 μs. An 8-bit frame was captured every two seconds for 150 seconds. Cell viability was assayed by stimulating the cells with 60 mM KCl at the end of the experiment. The changes in relative fluorescence unit (RFU) were calculated by selecting at least five regions of interest (ROI) from each experiment.

### Fluorescence confocal imaging

Cells were transfected with 1μg YFP tagged WT mAVPR2 receptors or mutant receptors, plus or minus 1μg mSCTR. 24 hrs post transfection, cells were rinsed with HBSS and fixed in paraformaldehyde at room temperature for 20 mins. They were then mounted using the Fluoro-Gel mounting medium (Electron Microscopy Sciences, Hatfield, PA). Fluorescence signal was measured on the same confocal microscope and objective. The machine was set to excite the samples at 514 nm and record 519–621 nm emission. The pinhole was set at 44μm and pixel dwell at 12.6 μs. Signals were recorded as 12-bit images. Surface to intracellular fluorescence ratio was calculated using the software ImageJ (NIH, US).

### Immunofluorescence staining

Samples preparation were the same as fluorescence confocal imaging until the mounting step. After fixation, samples were blocked with 1% BSA in PBST (PBS +0.1% Tween 20) for 30 min at room temperature. Samples were then incubated overnight at 4°C with 1:100 Anti-Calreticulin antibody [EPR3924]—ER Marker (Alexa Fluor® 488) (Abcam, Cambridge, MA). After three wash of PBS, Fluoro-Gel mounting were done before image acquisition.

### Statistical analysis

Statistical analysis and graph plotting were done by the computer software PRISM (version 5.03; GraphPad, San Diego, CA). All data were presented as means ± SEM from at least three independent experiments, each in duplicate or triplicate. Data were analyzed based on the assumption that the sample data followed a normal distribution. One-way ANOVA followed by a Dunnett’s test was used to compare experimental means against the control means for significance levels. Saturation BRET curves were fitted using the one-site total binding model. Dose response curves were fitted using the agonist stimulation model (three parameters) and values for maximal response (E_max_) and the half maximal effective concentration (EC_50_) were obtained from the curves.
